# Temporal induction of *Lhx8* by optogenetic control system for efficient bone regeneration

**DOI:** 10.1186/s13287-021-02412-8

**Published:** 2021-06-10

**Authors:** Delan Huang, Runze Li, Jianhan Ren, Haotian Luo, Weicai Wang, Chen Zhou

**Affiliations:** Hospital of Stomatology, Guanghua School of Stomatology, Guangdong Provincial Key Laboratory of Stomatology, Sun Yat-sen University, 56 Lingyuanxi Road, Guangzhou, 510055 China

**Keywords:** Optogenetics, *Lhx8*, BMSCs, Osteogenic differentiation, Bone regeneration

## Abstract

**Background:**

The spatiotemporal regulation of essential genes is crucial for controlling the growth and differentiation of cells in a precise manner during regeneration. Recently, optogenetics was considered as a potent technology for sophisticated regulation of target genes, which might be a promising tool for regenerative medicine. In this study, we used an optogenetic control system to precisely regulate the expression of *Lhx8* to promote efficient bone regeneration.

**Methods:**

Quantitative real-time PCR and western blotting were used to detect the expression of *Lhx8* and osteogenic marker genes. Alkaline phosphatase staining and alizarin red staining were used to detect alkaline phosphatase activity and calcium nodules. A customized optogenetic expression system was constructed to regulate *Lhx8*, of which the expression was activated in blue light but not in dark. We also used a critical calvarial defect model for the analysis of bone regeneration in vivo. Moreover, micro-computed tomography (micro-CT), three-dimensional reconstruction, quantitative bone measurement, and histological and immunohistochemistry analysis were performed to investigate the formation of new bone in vivo.

**Results:**

During the osteogenic differentiation of BMSCs, the expression levels of *Lhx8* increased initially but then decreased thereafter. *Lhx8* promoted the early proliferation of BMSCs but inhibited subsequent osteogenic differentiation. The optogenetic activation of *Lhx8* in BMSCs in the early stages of differentiation by blue light stimulation led to a significant increase in cell proliferation, thus allowing a sufficient number of differentiating BMSCs to enter the later osteogenic differentiation stage. Analysis of the critical calvarial defect model revealed that the pulsed optogenetic activation of *Lhx8* in transplanted BMSCs over a 5-day period led to a significant increase in the generation of bone in vivo.

**Conclusions:**

*Lhx8* plays a critical role in balancing proliferation and osteogenic differentiation in BMSCs. The optogenetic activation of *Lhx8* expression at early stage of BMSCs differentiation led to better osteogenesis, which would be a promising strategy for precise bone regeneration.

**Supplementary Information:**

The online version contains supplementary material available at 10.1186/s13287-021-02412-8.

## Background

Mesenchymal stem cells (MSCs) from different sources are multi-lineage cells with multi-directional differentiation potential and the ability of self-renewal. MSCs had been proved by multiple studies to play an important role in cell therapy and regenerative medicine [1, 2]. The osteogenic differentiation of MSCs forms the basis of bone tissue genesis and formation and can be divided into several stages [3, 4]: (1) cell proliferation; (2) cell differentiation and the expression of early osteogenic marker genes, including alkaline phosphatase (*ALP*), runt-related transcription factor 2 (*Runx2*), and osterix (*OSX*); (3) terminal mineral deposition accompanied by the expression of late osteogenic marker genes, including osteocalcin (*OCN*), osteopontin (*OPN*), and collagen type I (*Col-1*).

Bone regeneration strategies for medical application aim to develop methods that could precisely regulate osteogenic differentiation of MSCs. However, the fate of MSCs, including osteogenic differentiation, was determined by a complex network of transcription factors, growth factors, and non-coding RNAs [3, 5, 6]. Frequently used intervention strategies such as gene knockout, gene mutation, and chemical inhibitors were not able to regulate gene expression spatiotemporally for the sake of precise bone regeneration. Optogenetics was a powerful tool for the spatiotemporal regulation of biological activities in neuronal cells and has been widely used in other fields of biomedicine [7, 8]. This technology provides us with a method that can be used to regulate gene expression in a very precise manner, in which the intracellular signaling pathways associated with photoactivatable proteins are controlled by optical signals [9]. In a previous study, Bugaj and colleagues used light to activate the canonical Wnt/β-catenin signaling and achieved a higher rate of transcription than the natural ligand Wnt3a, thus demonstrating the feasibility of using optogenetics to regulate signaling pathways [10]. Wang et al. further showed that optogenetics cannot only realize gene regulation at the cellular and subcellular levels, but also permit expression at specific time points within the body [11]. Compared with other methods, optogenetics had less impact on the overall biological function of cells and was capable to manipulate gene expression in a quantitative and controllable manner without toxicity. In another study, Shao et al. used light to regulate the expression of target endogenous genes and successfully induced pluripotent stem cells to differentiate efficiently into specific cells with nervous function [12]. Collectively, these studies have shown that optogenetics, and the optical control of light-activatable proteins in cells, can provide us with the opportunity to precisely regulate gene expression with high levels of efficiency, sensitivity, and reversibility [11–13].

However, little is known about the application of optogenetics in the field of regenerative medicine [13]. In this study, an optogenetic regulation system was selected to regulate the target gene during osteogenesis. FKF1 (flavin-binding, kelch repeat, f-box 1) is a blue light receptor with a LOV domain (light, oxygen, or voltage) that binds to its ligand GIGANTEA (GI) when irradiated with blue light [14]. The LOV domain was first discovered in plant phototropins and was known to bind with FMN (flavin mononucleotide) in mammalian cells under blue light to open the light cycle. When light is lost, the cycle returns to the ground state [15]. Gal4 is a type of yeast transcription factor; the DNA-binding domain of Gal4 binds to the UAS sequence in an autonomous manner but activates transcription with the required participation of a transactivation domain [16]. VP16 is a powerful transactivation domain that can recruit basal transcription factors, but will not work in the absence of a DNA binding domain [17]. In a previous study, a light-activated expression system was constructed by fusing the LOV domain to VP16 and by fusing GI to Gal4 [8]. Based upon these findings, we optimized the optogenetic control system to precisely regulate specific target gene (*Lhx8*) for bone regeneration. In the current study, we adapted the illumination device and the light stimulation conditions, as well as investigated the application of this optogenetic control system in vivo.

LIM Homeobox 8 (*Lhx8*) was abundantly expressed during craniofacial development including bone development [18]. *Lhx8* played an important role in the self-renewal and proliferation potential of palatal mesenchymal cells and guaranteed normal palatal bone development by negatively regulating the expression of the cell cycle inhibitor *P57* (*Kip2*) in the anterior palatal region [19]. Previous studies have also shown that *Lhx8* was predominantly expressed in ectodermal mesenchyme and tooth germ mesenchyme derived from the neural crest; *Lhx8* was highly expressed during the early periods of tooth development but the expression level decreased during the later stages [18, 20]. Both of our previous study and another study indicated that the overexpression of *Lhx8* promoted the proliferation of human dental pulp-derived mesenchymal stem cells (DPSCs) while inhibiting odontogenic differentiation and negatively regulating the differentiation and maturation of mesenchymal tissue [20, 21]. This suggested that *Lhx8* might play an important role in regulating the balance between proliferation and differentiation in MSCs. We had also confirmed that *Lhx8* can significantly promote the proliferation of bone marrow mesenchymal stem cells (BMSCs) [13]. However, the specific function of *Lhx8* in the process of osteogenic differentiation in BMSCs has yet to be elucidated. Therefore, further studies from the perspective of dynamic gene regulation, for example, *Lhx8*, were of great significance in increasing our understanding of how MSCs was determined and participated in bone regeneration.

In this study, we investigated the expression pattern and function of *Lhx8* during the process of osteogenic differentiation in BMSCs. By constructing an optogenetic expression system to precisely regulate the expression of *Lhx8*, we successfully improved osteogenesis and new bone formation by BMSCs, both in vitro and in vivo.

## Methods

### Isolation and characterization of BMSCs

All procedures involving animals were approved by the Institutional Animal Care and Use Committee of Sun Yat-Sen University. Femurs and tibias were collected from SD rats (4–6 weeks of age) under aseptic conditions. Bone marrow cells were harvested by flushing with a syringe. Separated cells were then cultured as described previously [22, 23]. Cells from passages 2 to 3 (P2 –3) were used for experiments. After passaging 2–3 times, the BMSCs were analyzed by multilineage differentiation assays and flow cytometry (Figure S1, Additional file 2). In brief, we detected the expression levels of CD34, CD45, CD29, CD44, and CD90 by a BD FACscalibur (BD Biosciences, USA). Isotype controls were used as negative controls. The osteogenic, lipogenic, and chondrogenic differentiation ability of the BMSCs was confirmed by alizarin red staining after 21-day culture, oil red staining after 14-day culture, and alcian blue staining after 21-day culture in different induction mediums, respectively.

### Plasmid construction, virus packaging, and infection

The coding sequences of GI-Gal4DBD, LOV-VP16, and *Lhx8* were cloned into the lentiviral expression vector pWPI, resulted in the creation of following constructs: pWPI-GI-Gal4DBD, pWPI-LOV-VP16, and pWPI-Lhx8. A pWPI-5×Gal4 UAS-Lhx8 construct was synthesized by replacing the EF1α promoter of pWPI-Lhx8 with the 5×Gal4 UAS promoter. All plasmid synthesis and modification were performed by Genscript (Nanjing, China). The sequences used are shown in Additional file 8. A Calcium Phosphate Transfection Kit (Invitrogen, USA) was used, in accordance with the manufacturer’s instructions, to transfect HEK293T cells with 5×Gal4 UAS-Lhx8, Lhx8 or Control pWPI, GI-Gal4DBD, LOV-VP16 in pWPI vector, pMD2.G, and psPAX2 vectors to generate lentiviral particles. The supernatant, containing corresponding lentiviral particles, was collected for two consecutive days after transfection, filtered by a 0.45-μm filter, and then purified by a Lentivirus Purification Kit (Cell Biolabs, USA). BMSCs or HeLa cells were transfected with lentivirus in 8μl/ml of polybrene (Santa Cruz Biotechnology, USA) at a multiplicity of infection (MOI) of 10 IU/cell.

### Optogenetic stimulation

The cells were seeded onto a culture dishes or plates at a density of 5×10^4^/cm^2^ and then optically stimulated at 48h after transfection. According to our previous study [13], light irradiance was performed at 1.0 mW/cm^2^ for a specified period using a custom LED light device (Figure S3 a, Additional file 4). The local light intensity was adjusted through the distance between light source and cells. Untreated samples were placed in a continuous dark environment. Light sources of different wavelengths were selected according to the requirements of experiment. For *in vivo* experiments, rats were maintained in cage and illuminated from above by a blue LED lamp (100 mW/cm^2^) for 1 h a day, or left in the dark (Fig. 4a). During the experiment, the intensity of light stimulation was measured by an optical power meter (Q8230; Advantest, Japan).

### Osteogenic induction, alizarin red, alkaline phosphatase staining, and quantification

Osteogenic differentiation of BMSCs was induced using osteogenic induction medium consisting of a 10-mM β-glycerol phosphate (Sigma, USA), 0.1μM of dexamethasone (Sigma, USA), and 50 mg/mL of ascorbate-2-phosphate (Sigma, USA); the medium was refreshed every 3 days. For alkaline phosphatase (ALP) staining, the BMSCs were fixed in 4% paraformaldehyde, washed with phosphate buffered solution (PBS), and stained according with the Alkaline Phosphatase Staining Kit in accordance with the manufacturer’s instructions (Yeasen, China). Stained cells were observed and photographed by a phase-contrast microscope. ALP activity was quantified by a commercial ALP kit (Jiancheng, Nanjing, China). We also collected supernatant and cell lysates. Cells were incubated with p-nitrophenyl phosphate solution, and then, alkaline phosphatase activity was calculated by detecting absorbance at 520 nm. Quantification was conducted by dividing the absorbance by the protein concentration, as determined by the BCA protein assay (Beyotime, China). After 21 days of culture in osteogenic medium, we used Alizarin Red S solution (Cyagen Biosciences, China) to identify areas of calcium deposition. In brief, BMSCs were fixed by 4% paraformaldehyde, stained in Alizarin Red S solution at room temperature for 10 min, and then washed in phosphate-buffered solution (PBS). Stained cells were then observed and photographed by a phase-contrast microscope. The semi-quantification of Alizarin Red S concentrations was conducted by a quantitative de-staining procedure using 10% cetylpyridinium chloride (CPC) (Sigma, USA) for 15 min at room temperature, and absorbance was measured in a microplate spectrophotometer (Bio-Tek, UK) at 562 nm.

### qPCR

Cells samples were lysed by TRIzol (Invitrogen, USA) for total RNA extraction. Then, total RNA was reverse transcribed into cDNA with the Prime Script RT Master Mix Kit (TaKaRa, Japan) in accordance with the manufacturer’s instructions. Real-time qPCR was performed in triplicate in 20μl reactions containing SYBR Green master mix (Roche, USA). Primer sequences are shown in Table S1 (see Additional file 1), and the relative expression of genes was calculated in accordance with the manufacturer’s instructions.

### Western blotting

Total protein was extracted by RIPA lysis buffer (CWBIOTECH, China) and concentration was determined by the BCA protein assay (Beyotime, China). Proteins were then loaded and separated by SDS-PAGE (Genscript, China) and transferred to a polyvinylidene fluoride (PVDF) membrane (Millipore, USA) with a wet transfer blotting system (Bio-Rad, CA). After blocking for 1.5h with 5% bovine serum albumin (Biofroxx, Germany), the membranes were incubated at 4°C overnight with appropriate primary antibodies:monoclonal rabbit anti-Lhx8 antibody (1:1000, Abcam, ab137036), polyclonal rabbit anti-Runx2 antibody (1:1000, Abcam, ab23981), polyclonal rabbit anti-ALP antibody (1:1000, Abcam, ab83259), polyclonal rabbit anti-OSX antibody (1:1000, Abcam, ab22552), polyclonal rabbit anti-OCN antibody (1:1000, Abcam, ab93876), polyclonal rabbit anti-OPN antibody (1:1000, Abcam, ab8448), polyclonal rabbit anti-Col-1a antibody (1:1000, Abcam, ab34710), and monoclonal rabbit anti-GAPDH antibody (1:1000, Cell Signaling, #5174s). The following morning, membranes were washed and then incubated with relevant secondary antibodies (1:1000, Beyotime, China) for 1h at room temperature. The intensity of stained bands were measured by chemiluminescence with the ECL western blotting substrate kit (Millipore, USA). The relative intensities of each immunoreactive protein were then quantified by ImageJ 1.8.0 software (NIH, USA).

### CCK-8 assays

Cell viability was analyzed by the CCK-8 kit (Vazyme, China). BMSCs were seeded in 96-well plates and cultured with appropriate treatments. In brief, 100μl of fresh culture medium containing 10% of CCK-8 solution was gently added into each well. After 2h of incubation, optical density values were read at 450nm using a microplate spectrophotometer (Bio-Tek, UK).

### EdU staining

A 5-ethynyl-20-deoxyuridine (EdU) incorporation assay was used to label the cells in S-phase of the cell cycle. BMSCs were seeded onto glass coverslips, incubated with 50μM of EdU (Keygen, China) for 1h, then fixed and permeabilized according to the manufacturer’s instructions. After incubation with kFlour488 Click-iT dye-conjugate cocktail for 30min, the cells were counterstained with Hoechst 33342. Finally, the samples were observed and photographed by a laser scanning confocal microscope (Zeiss, Germany).

### Construction and characterization of PLGA scaffolds

Poly(lactic-co-glycolic acid) (PLGA) porous scaffolds were synthesized as described previously [13]. In brief, 1g of PLGA (75:25, Sigma-Aldrich, USA) was dissolved in 16 ml of dichloromethane (DCM). Then, 9 g of sodium chloride salt (NaCl), with a diameter ranging from 125 to 300μm was added into the PLGA/DCM solution. After mixing, the solution was poured into a Petri dish and placed in a chemical hood to dry. The dried polymer was then soaked in deionized water for 72h and cut into PLGA discs at a diameter of 5mm. Discs were then sterilized by UV irradiation and immersed in the medium. A 50μl of cell suspension (at density of 1×10^7^/ml) was then dropped onto the scaffold. The morphology of the scaffolds and cell adhesion on the scaffolds was then observed by scanning electron microscopy (SEM) (Figure S5 a-f, Additional file 6). Cell viability on the scaffold was investigated by CCK-8 assays (Figure S5 g, Additional file 6). In addition, the BMSCs-PLGA scaffolds were transplanted in vivo after being cultured overnight in osteogenic medium.

### The critical calvarial defect model and implantation of the BMSC-PLGA scaffold

All animal procedures were conducted under the guidance of the Care and Use of Laboratory Animals of Sun Yat-Sen University and approved by Institutional Animal Care and Use Committee (IACUC) of Sun Yat-Sen University (Reference: SYSU-IACUC-2018-000041). A rat critical calvarial defect model was created in vivo. Eight-week-old male SD rats, weighing 250–300g, were randomly divided into five groups: (1) Ctrl, *n*=3; (2) Ctrl+light, *n*=4; (3) opto Lhx8+dark, *n*=3; (4) opto Lhx8+ light, *n*=3; and (5) over Lhx8, *n*=3. Rats were anesthetized with 1% pentobarbital (0.4 ml/100g) via intraperitoneal injection. Prior to surgery, the skin at the surgical site was cleaned and sterilized. Then, incisions of approximately 2 cm in length were made along the midsagittal line in the skull. Next, we created 5-mm circular full-thickness bone defects, as described previously [13, 24]. Rats were given different transplants, as appropriate, and the incisions were then closed. The rats were subsequently subjected to blue light stimulation, as indicated. After 8 weeks, the rats were euthanized, and bone tissues (including transplants) were harvested. Tissues were fixed in 4% paraformaldehyde solution for 24h and stored in 75% ethanol to await subsequent analysis.

### Micro-CT analysis

High-resolution micro-CT scanning and analysis were performed by a micro-CT scanner (Scanco Medical μCT 50, Switzerland). After being placed in holders, the samples were scanned at 70kVp and 114μA. Three-dimensional (3D) reconstruction was carried out using data analysis software (Avizo 8.1, USA). The region of interest was set by a 5-mm circle along the defect edge and then used to calculate the ratio of bone volume (BV) to total volume (TV) and bone mineral density (BMD) so that we could quantitatively assess bone formation.

### Histological and immunohistochemistry analysis

The samples were fixed in 4% paraformaldehyde solution for 24h, decalcified in 0.5 mol/L ethylene diamine tetraacetic acid (EDTA) for 6 weeks. After dehydrated and embedded in paraffin, the samples were cut into 4-μm sections for staining. For histological analysis, the sections were stained with hematoxylin and eosin (HE) as well as Masson’s trichrome stain according to the manufacturer’s protocol (Servicebio, China). For immunohistochemistry analysis, the sections were incubated with primary antibodies at 4°C overnight after dewaxed, rehydrated, and antigen retrieval. The primary antibodies were as follows: polyclonal rabbit anti-OCN antibody (1:200, Abcam, ab93876) and polyclonal rabbit anti-OPN antibody (1:200, Abcam, ab8448). After incubated with the corresponding secondary antibodies (Invitrogen, USA) for 30 min at room temperature, the signals were detected by diaminobenzidine (DAB) (Servicebio, China) and counterstained with hematoxylin (Servicebio, China). Finally, all tissue slices were photographed by the Aperio AT2 slide scanner (Leica Biosystems, Germany). The immunohistochemistry images were assessed by ImageJ with the IHC-Toolbox plugin (NIH, USA).

### Statistical analysis

Data are expressed as mean ± standard deviation unless indicated otherwise. Comparisons were performed with GraphPad Prism 8.0 software using the Student’s two-tailed *t* test or one-way analysis of variance (ANOVA). Differences between groups or treatments are given as ns (non-significant) or significant (*P<0.05, **P<0.01, ***P<0.001, and ****P<0.0001). All experimental values were obtained from at least three independent biological repetitions.

## Results

### Dynamic expression of *Lhx8* during osteogenesis in BMSCs

**Fig. 1:**
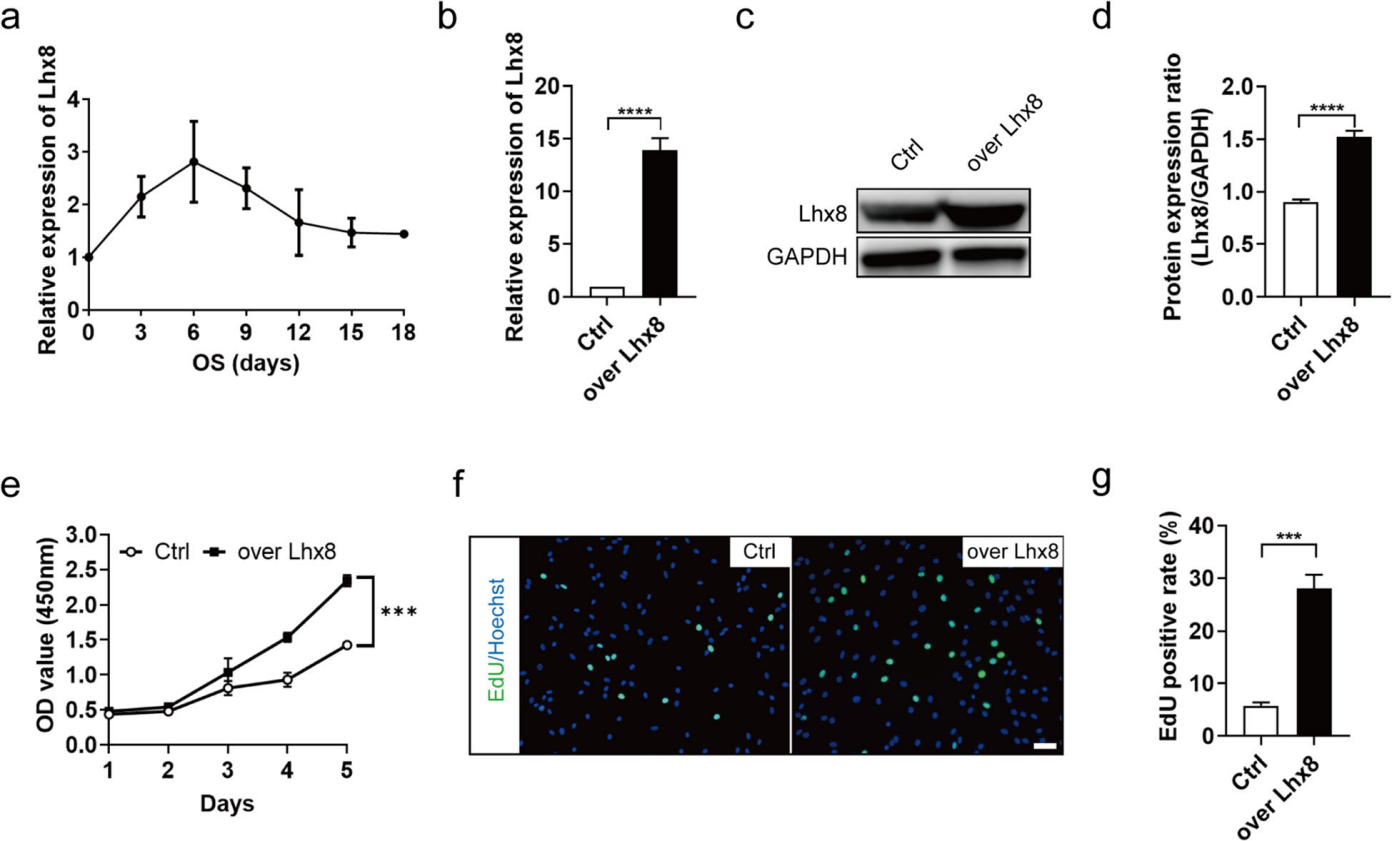
*Lhx8* showed dynamic patterns of expression during osteogenic differentiation and promoted cell proliferation. **a** Relative mRNA expression of *Lhx8* during the osteogenic induction of rat BMSCs in vitro. **b** qPCR analysis showed that the expression of *Lhx8* mRNA was significantly increased in the over Lhx8 group. BMSCs transfected with lentivirus overexpressing *Lhx8* and control respectively. **c**, **d** The protein expression of *Lhx8* was evaluated by western blotting. **e** CCK-8 growth curves showed that *Lhx8* promoted the cell viability of BMSCs. **f**, **g** Representative images and quantitative analysis of EdU staining (green) of BMSCs in the two groups. Nuclear DNA was counterstained with Hoechst. Scale bar=50μm. All experiments were performed in triplicates. ***P<0.001, ****P<0.0001 vs*.* Ctrl

To investigate the characteristics of *Lhx8* expression in BMSCs during osteogenesis, we first extracted BMSCs from the femurs and tibias of SD rats, which was then identified by morphology, multilineage differentiation ability, and cell surface markers as depicted in Supplementary Figure 1. After induction for different periods in osteogenic differentiation medium, the transcript levels of *Lhx8* in BMSCs were detected for 18 days period (Fig. 1a). The expression pattern of *Lhx8* increased from day 0 and peaked at day 6 and then gradually declined to almost baseline level, indicating a dynamic expression pattern of *Lhx8* during the process of osteogenesis. This result that high levels of *Lhx8* were expressed durin*g* early stage of osteogenesis indicated its function in cell viability.

### *Lhx8* promoted the proliferation of BMSCs

We transfected BMSCs with an engineered lentivirus overexpressing *Lhx8* and an empty control virus as a control. Next, we confirmed the efficiency of this overexpression by qPCR and western blotting. These analyses showed that the mRNA and protein levels of the Lhx8 overexpression group (over Lhx8) were significantly higher than those of the control group (Ctrl) (Fig. 1b–d). CCK-8 growth curves showed that *Lhx8* promoted the cell viability of BMSCs compared with control (Fig. 1e). EdU immunofluorescence staining also indicated that the overexpression of *Lhx8* significantly promoted the proliferation of BMSCs (Fig. 1f–g).

### Sustained expression of *Lhx8* inhibits osteogenic differentiation of BMSCs

**Fig. 2:**
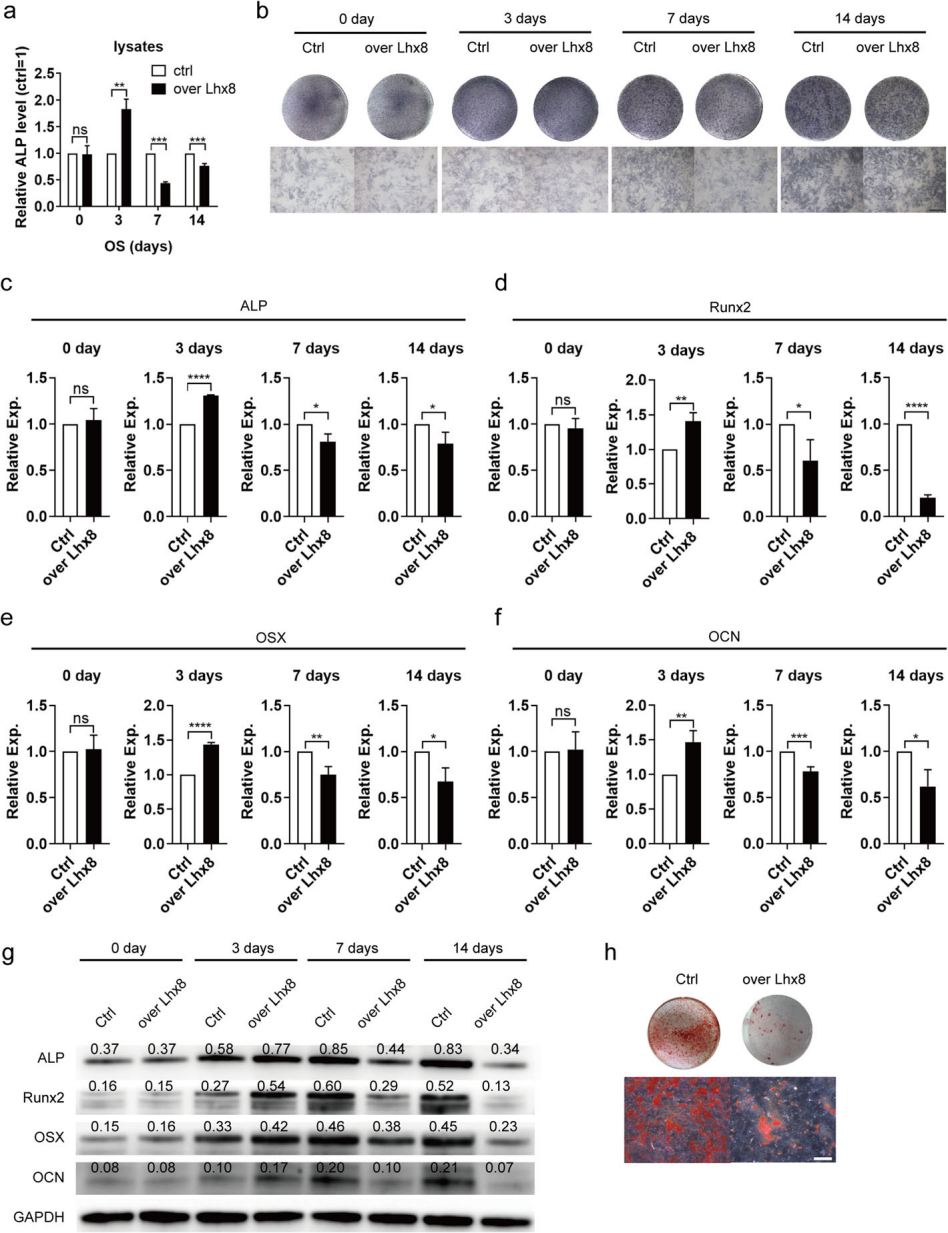
The sustained expression of *Lhx8* inhibited the osteogenic differentiation of BMSCs. **a** ALP activity was detected by colorimetric assay in the cell lysates of BMSCs on day 0, 3, 7, and 14 under osteogenic induction. **b** Gross appearance of ALP staining is shown in the upper image while the microscopic appearance is shown in the lower image. Scale bar=400μm. **c–f** The mRNA expression of osteogenic-specific genes (*ALP*, *Runx2*, *OSX*, *OCN*) after osteogenic induction on days 3, 7, and 14 day. **g** The protein expression of osteogenic-specific genes (*ALP*, *Runx2*, *OSX*, *OCN*) after osteogenic induction on day 0, 3, 7, and 14. The quantitative western blot results were shown above the bands. **h** Gross appearance of Alizarin red staining performed after 21 days of osteogenic induction is shown in the upper image while the microscopic appearance is shown in the lower image. Scale bar=400μm. All experiments were performed in triplicate. *P<0.05, **P<0.01, ***P<0.001, ****P<0.0001 vs. Ctrl

To further investigate the role of *Lhx8* in the osteogenic differentiation of BMSCs, we analyzed ALP activity in culture medium supernatants and cell lysates produced from the over Lhx8 and Ctrl groups on day 0, 3, 7, and 14 after osteogenic induction. Analysis showed that there was no significant difference in ALP activity in the supernatant of these two groups when measured at different timepoints (Figure S2 a, Additional file 3). However, we detected significant differences in the ALP activity of the cell lysate (Fig. 2a). ALP activity in the cell lysate of the over Lhx8 group was lower than that of the Ctrl group on day 7 and 14, but was higher on day 3. Results of ALP staining (Fig. 2b) were consistent with those arising from the quantitative analysis of the cell lysate. We also tested the mRNA and protein expression levels of osteogenic-specific genes related to osteogenic differentiation after 0, 3, 7, and 14 days of osteogenic induction (Fig. 2c–g, Figure S2 b-j, Additional file 3). The mRNA and protein levels of *ALP* at four different time points were consistent with the trend shown in the staining results (Fig. 2c, g, Figure S2 e, Additional file 3). The mRNA and protein levels of *Runx2*, *OSX*, *OCN*, and *OPN* followed similar trends in that the overexpression of *Lhx8* promoted the expression of these bone formation-related genes on day 3 but inhibited them on day 7 and 14 (Fig. 2d–g, Figure S2 b, d, f-i, Additional file 3). Notably, the expression pattern of *Col-1a* was different from other genes (Figure S2 c-d, j, Additional file 3). The difference of *Col-1a* between two groups was statistically significant only on day 14 of osteogenic induction. Next, we stained BMSCs with Alizarin Red S after 21 days of osteogenic induction to detect the amount of calcium mineralized nodules. The results showed that overexpression of Lhx8 significantly inhibited the formation of calcium nodules (Fig. 2h). Overall, these results indicated that *Lhx8* played different roles at different stages during the process of osteogenic differentiation of BMSCs.

### Establishment of an optogenetic system for the temporal control of *Lhx8* expression in BMSCs

**Fig. 3:**
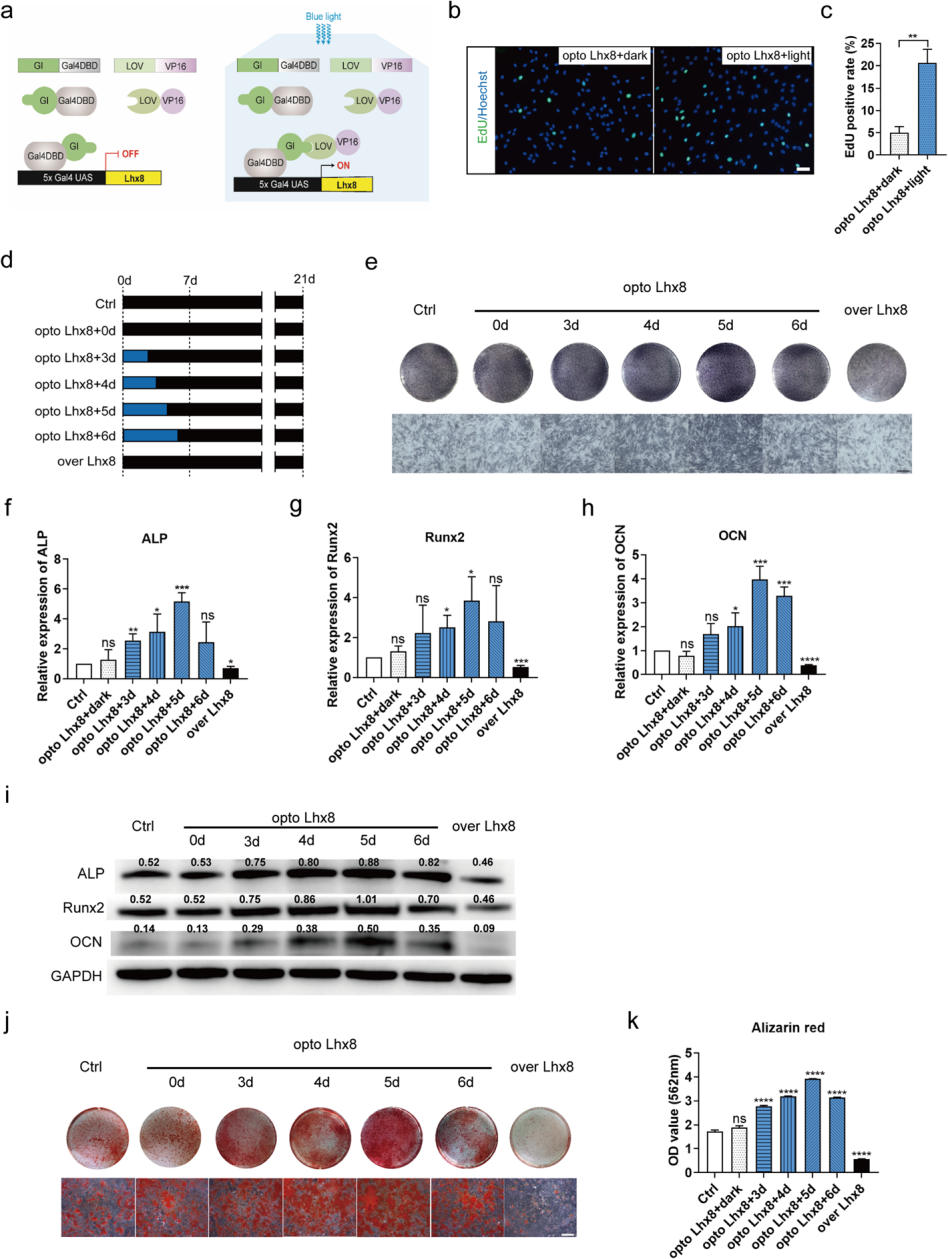
An optogenetic control system for the optimization of *Lhx8* expression towards efficient osteogenesis in vitro*.* **a** Schematic representation of the optogenetic regulation system for *Lhx8*. Transactivation of *Lhx8* was activated by blue light stimulation. **b**, **c** Representative images and quantitative analysis of EdU staining (green) of BMSCs in the two groups. Nuclear DNA was counterstained with Hoechst. Scale bar=50μm. **d** Schematic representation of grouping and the experimental procedure. **e** Representative images of gross appearance (upper) and microscopic images (lower) with ALP staining after 7 days of osteogenic induction. Scale bar=300μm. **f**–**h** The mRNA expression of osteogenic-specific genes (*ALP*, *Runx2*, *OCN*) after osteogenic induction on day 7. **i** The protein expression of osteogenic-specific genes (*ALP*, *Runx2*, *OCN*) after osteogenic induction on day 7. The quantitative western blot results were shown above the bands. **j** Representative images of gross appearance (upper) and microscopic images (lower) with Alizarin red S staining of calcified nodules after 21 days of osteogenic induction. Scale bar=100μm. **k** Semi-quantitative evaluation of Alizarin Red S staining by CPC. All experiments were performed in triplicates. *P<0.05, **P<0.01, ***P<0.001, ****P<0.0001 vs. Ctrl

According to the above findings, we hypothesized that Lhx8 promoted proliferation in the early stage of osteogenesis in BMSCs but inhibited osteogenic differentiation in the later stage of osteogenesis. We found that the effect of *Lhx8* on BMSCs’ biological function changed during days 3 to 7 after osteogenic induction. In order to regulate the expression of *Lhx8* precisely, we engineered an optogenetic regulation system that could activate *Lhx8* expression in the presence of blue light but not in the dark (Fig. 3a). In brief, cells were infected with lentiviruses expressing GI-Gal4DBD and LOV-VP16 driven by EF1α; *Lhx8* was driven by 5×Gal4 UAS in the pWPI vector. We verified the light expression system and optimized the light conditions in HeLa cells and BMSCs (Figure S3, Additional file 4). Based on our previous studies, we selected light intensity at 1 mW/cm^2^ for all in vitro experiments. An adjustable LED light device was used to stimulate the cells with light (Figure S3 a, Additional file 4). As expected, expression of Lhx8 in HeLa cells transfected with this specific optogenetic regulation system (opto Lhx8) was activated by blue light, although there was also a small amount of *Lhx8* leakage under green light (Figure S3 b, Additional file 4). Continuous blue light irradiation had no effect on the cell viability of BMSCs, suggesting that blue light treatment caused no significant damage to the cells (Figure S3 c, Additional file 4). Results of EdU assay also indicated that blue light treatment caused no significant damage to the proliferation of BMSCs (Figure S3 d-e, Additional file 4). After 30min of illumination, the expression of *Lhx8* mRNA in the opto Lhx8 group of HeLa cells increased gradually, peaked at 12h, and then returned to the baseline level after 24h (Figure S3 f-g, Additional file 4). We performed pulse stimulation for different duration and found that the best activation was achieved by 30min of illumination (Figure S3 h, Additional file 4). In addition, EdU assay showed that blue light stimulation promoted the proliferation of BMSCs with opto Lhx8 system (Fig. 3b–c).

Based on these results, we designed and carried out an investigation to identify the special role of *Lhx8* during osteogenesis in BMSCs. The expression of *Lhx8* was stimulated by blue light for different days during the early stages of osteogenesis in BMSCs with the aim to promote the cell proliferation stage (Fig. 3d). The experimental groupings and process are shown in Fig. 3d. ALP staining on day 7 of osteogenic induction showed that the ALP activity of the opto Lhx8 groups under light stimulation was higher than the control, with that of the opto Lhx8 + 5d group being highest (Fig. 3e). We also detected the mRNA and protein levels of osteogenic-specific genes and identified similar patterns of *ALP*, *Runx2*, and *OCN* (Fig. 3f–i, Figure S4, Additional file 5). On day 21 of osteogenic induction, Alizarin Red S staining results indicated that the cells received 5 days of blue light illumination produced more calcium nodules (Fig. 3j), which was also confirmed by semi-quantitation of Alizarin Red staining (Fig. 3k). To this end, we found that the time-specific role of *Lhx8* during osteogenic differentiation of BMSCs probably changed at day 5 after osteogenic induction. The optogenetic activation of *Lhx8* expression by blue light illumination for 5 days after osteogenic induction presented better osteogenic effect. Collectively, these data suggested optogenetic activation of *Lhx8* at early stage of induction significantly promoted osteogenesis of BMSCs in vitro.

### Optogenetic activation of *Lhx8* during the early period of osteogenesis promoted bone regeneration in vivo

**Fig. 4:**
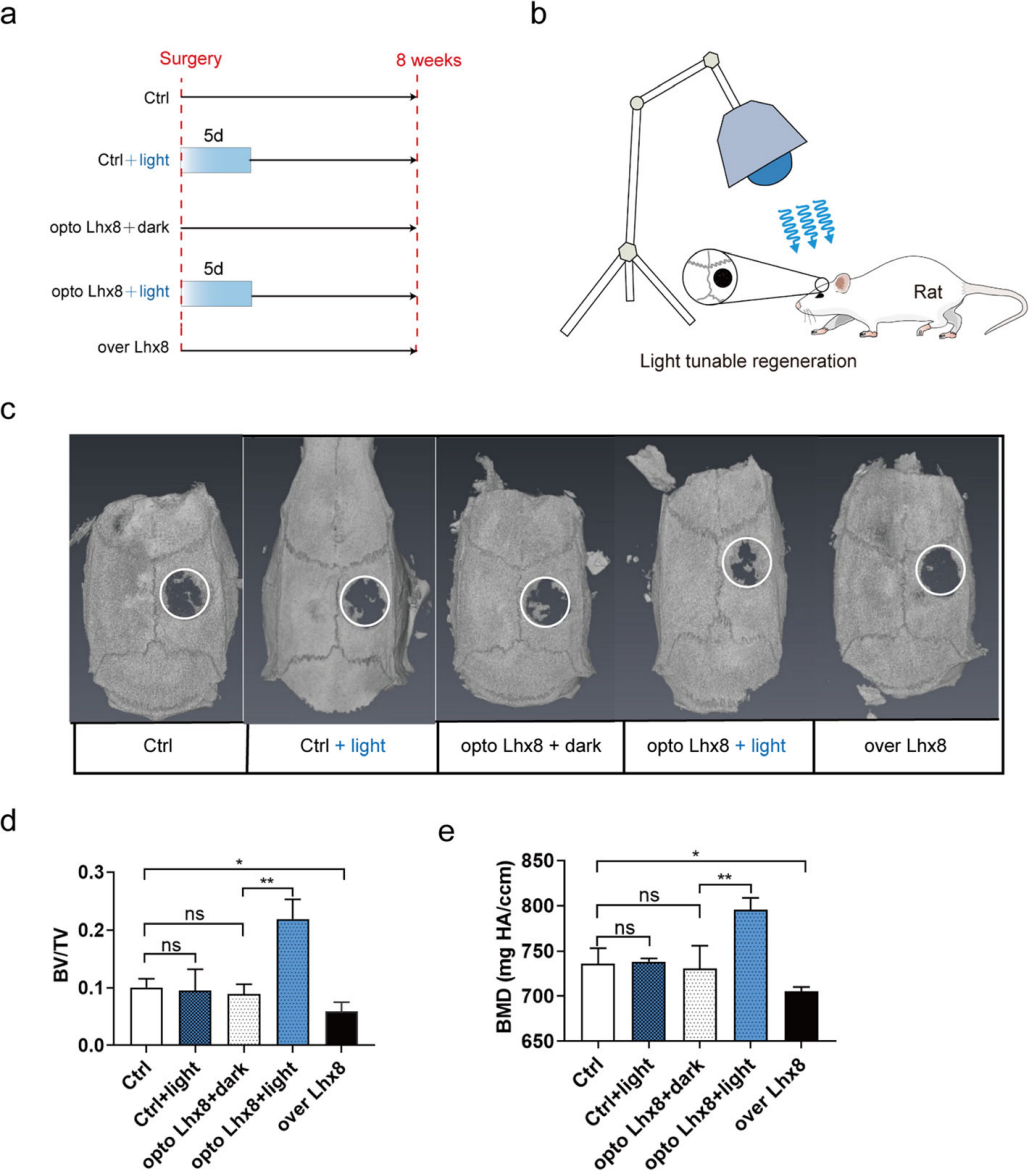
The optogenetic regulation of *Lhx8* for efficient bone regeneration in vivo. **a** Schematic diagram of animal light stimulation. **b** Schematic representation of the animal experimental procedure. **c** Representative images of 3D restruction showed the bone formation of BMSCs in vivo 8 weeks after surgery. *n*=3–4. **d** Quantitative analysis of bone formation by bone volume (BV)/total volume (TV) in specific regions of interest. **e** Quantitative analysis of bone formation by bone mineral density (BMD) in specific regions of interest. *n*=3–4. *P<0.05, **P<0.01 vs. Ctrl

**Fig. 5:**
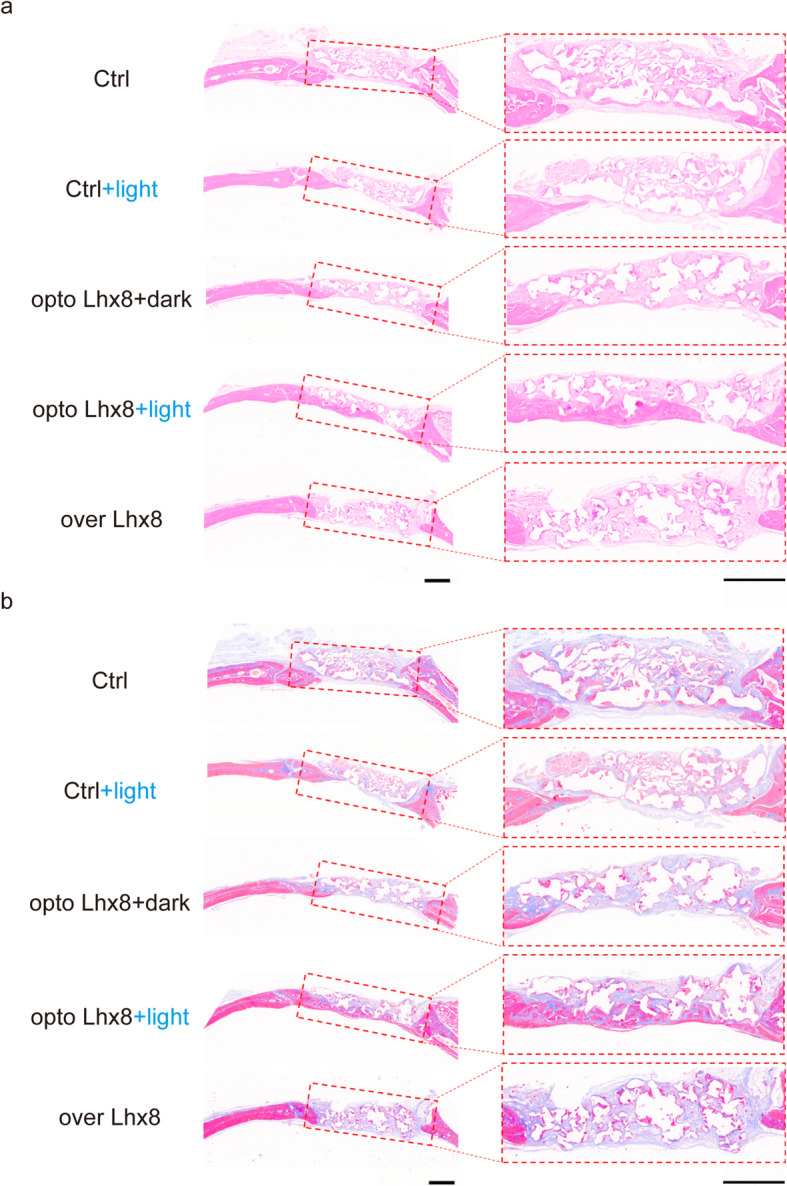
Histological analysis of bone regeneration in vivo. **a** Representative images of HE staining in each group. Scale bar=1mm. **b** Representative images of Masson’s trichrome staining in each group. Scale bar=1mm. *n* =3–4

**Fig. 6:**
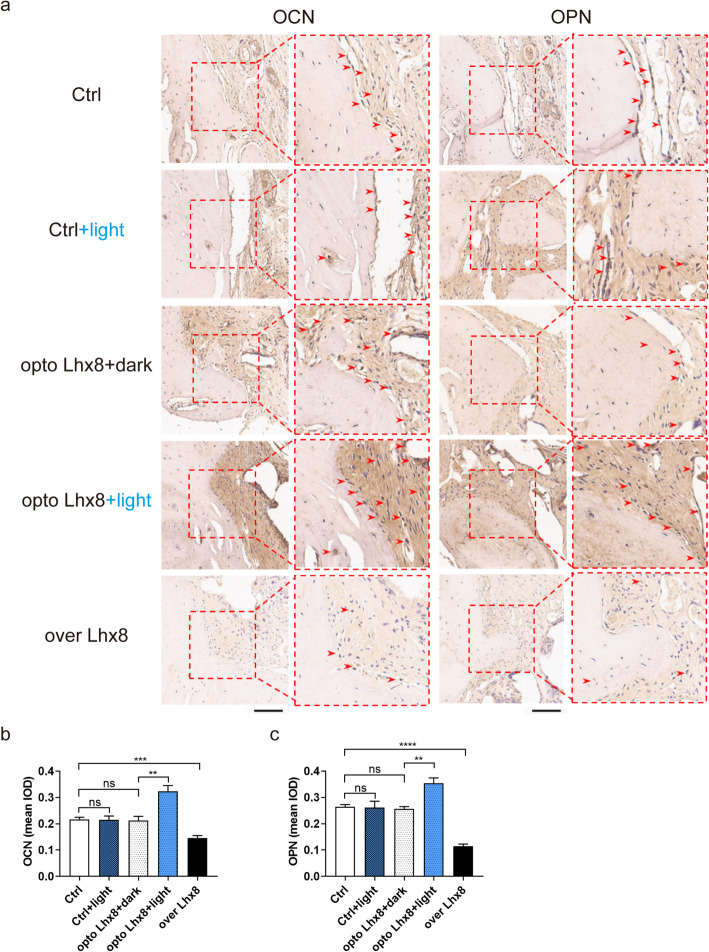
Immunohistochemistry of bone regeneration in vivo. **a** Representative immunohistochemical staining images of OCN and OPN. Arrows point to the positive area. Scale bar=100μm. **b**, **c** Quantitative analysis of OCN and OPN expression from immunohistochemical staining in **a**. IOD: immunohistochemical optical density. *n* =3–4. **P<0.01, ***P<0.001, ****P<0.0001 vs. Ctrl

In order to verify that our hypothesis was valid during the osteogenesis of BMSCs in vivo, we constructed a critical calvarial defect model using SD rats. PLGA was chosen as the synthetic material of the scaffold because of its biocompatibility and low toxicity. PLGA scaffolds were cut into a shape that was consistent with the size of the bone defect site (Figure S5 a, Additional file 6) and because its porous structure was conducive to the adhesion of BMSCs without affecting cell viability (Figure S5 b-g, Additional file 6). Optogenetic activation of *Lhx8* by blue light effective in BMSCs with opto Lhx8 system in vivo (Figure S6, Additional file 7). According to the results of in vitro experiments, we randomly divided the animals into five groups: Ctrl, Ctrl + light, opto Lhx8 + dark, opto Lhx8+ light, and over Lhx8 (Fig. 4a). After the BMSCs + PLGA scaffolds were transplanted into the defects, we performed blue light stimulations using an LED light source from above the rats (Fig. 4b). By comparing the 3D reconstruction, BV/TV ratio and BMD of samples from the Ctrl group and Ctrl + light group, we found that blue light stimulation itself had no significant effect on the bone formation of BMSCs in vivo (Fig. 4c–e). However, there were significant differences between the opto Lhx8 + dark group and the opto Lhx8 + light group in 3D reconstruction, BV/TV ratio, and BMD at the bone defect site. This suggested that the optogenetic regulation of Lhx8 by light illumination promoted the bone formation of BMSCs in vivo (Fig. 4c–e). Notably, the results of the over Lhx8 group revealed that the continuous expression of Lhx8 significantly inhibited the bone formation of BMSCs in vivo (Fig. 4c–e). These data suggested that the light-controlled expression of *Lhx8* during the early proliferation stage promoted osteogenesis of BMSCs in vivo. HE staining illustrated more new bone formation in opto Lhx8 + light group whereas less in over Lhx8 group, compared to other groups (Fig. 5a). Masson’s trichrome staining revealed more collagen fiber bundles arranged compactly in opto Lhx8 + light group (Fig. 5b). Furthermore, immunohistochemistry staining showed that protein expression levels of bone formation marker OCN and OPN were the highest in opto Lhx8 + light group (Fig. 6a). The quantitative analysis of immunohistochemical staining revealed significantly higher OCN and OPN expression in opto Lhx8 + light group compared to the other four groups (Fig. 6b, c). These results, derived in the critical calvarial defect model, were consistent with those from our in vitro experiments under osteogenic induction. In a word, it was confirmed that the optogenetic regulation of *Lhx8* promoted the bone formation by BMSCs, both in vitro and in vivo.

## Discussion

In this study, we investigated the expression profile of *Lhx8* in BMSCs during the process of osteogenic induction. Our analysis revealed the time-specific role of Lhx8 at different stages of the osteogenic differentiation process in BMSCs. *Lhx8* was considered to play a crucial role in development and the cell fate determination [18]. The dynamic expression pattern of *Lhx8* was closely related with its effect on cell function, especially the balance between proliferation and terminal differentiation, which was one of the critical factor of regeneration. Our previous study found that in the early stage of dental mesenchymal development and maturation, the robust expression of *Lhx8* activated Wnt and TGFβ signaling pathways, which was necessary to limit the premature differentiation of dental mesenchymal cells into odontoblasts [20]. While in the late stage, the decreased expression of *Lhx8* reduces the activation of Wnt and TGFβ, thereby meeting the requirements of dental mesenchymal differentiation and maturation [20]. In the current study, although dynamic expression pattern of Lhx8 in BMSCs during osteogenesis was similar to that in dental mesenchymal cells during dental mesenchymal development and maturation, it was necessary to further reveal the mechanism of Lhx8 dynamic expression and function by future studies.

During the process of osteogenic differentiation of MSCs, levels of multiple transcription factors, growth factors, and non-coding RNAs change accordingly over time, suggesting that these factors may play diverse roles [3, 5, 6]. A previous study showed that the spatiotemporal release of these vital factors promoted the osteogenic differentiation of MSCs in a better manner than continuous release [25]. Our current study of *Lhx8* provides a precedent for the research of other crucial factors.

The balance between proliferation and differentiation is an important issue that needs to be solved if we are to apply MSCs in tissue regeneration and stem cell therapy [2]. On the one hand, MSCs maintain a steady state of their own cell numbers via cell proliferation in vivo. On the other hand, MSCs can acquire the function of bone tissue by directly differentiating into osteoblasts and osteocytes [2]. In the process of osteogenic differentiation in MSCs, we should not only ensure that a sufficient number of cells are present, but also prevent the cell senescence caused by over-expansion which might impair cell proliferation and differentiation of MSCs [26]. Therefore, we utilized an optogenetic regulation system to control the expression of *Lhx8* within a limited period of time so that we could promote the proliferation ability of BMSCs without affecting their subsequent ability for osteogenic differentiation.

In order to achieve the temporal regulation of *Lhx8*, we designed an optogenetic regulation expression system for *Lhx8*. Based on previous studies, and the results of our current results, it was clear that *Lhx8* promoted the initial cell proliferation of MSCs during the process of bone formation, but inhibited subsequent cell differentiation. In our study, *Lhx8* was temporarily overexpressed by light stimulation during the early stages of osteogenesis to ensure temporal cell proliferation and permit a sufficient number of cells to be involved in BMSC bone formation. We also constructed a calvarial defect model and used this to prove that our system was safe and effective for in vivo applications. However, the regulation of a single gene (*Lhx8*) was not able to fully control the determination of fate for the MSCs. In a manner that is similar to development, the process of bone regeneration in MSCs arises from the spatiotemporal and sequential expression of multiple factors. Thus, in order to create optimal protocols for regeneration, it is important that we combine an increased number of key factors.

Optogenetics is a breakthrough technology that enables the sophisticated control of genetically encoded molecules. The optogenetic regulation expression system that we used in the present study was controlled by blue light which had obvious limitations as low penetration. This meant that the stimulating site must be close to the body surface; otherwise, we were not able to achieve effective expression efficiency. As optogenetic techniques had developed, many optimized systems have become available that are activated by near-infrared light or red light [12, 27]. Near-infrared light and red light are considered to be ideal sources of stimulation due to their higher penetration and reduced risk of photodamage. In our following research, we planned to engineer a more optimized control system to expand the scope of our applications for our system. Although the osteogenesis of MSCs does not require regulation as sophisticated as that of the directional differentiation of neuron cells, the precise regulation strategy proposed herein can be applied to the functional study of important genes and other methods for the regeneration of fine tissue, such as periodontal regeneration. The periodontal complex includes cementum, the periodontal ligament, and alveolar bone. Due to its complex anatomy, the repair of periodontal tissue defects has always been a challenge. A recent study indicated that the same treatment had completely different effects on the regeneration of these different tissue components [28]. Therefore, the precise regulation of tissue specificity is expected to solve the obstacle of periodontal tissue regeneration. We could activate the associated parts of the specific portion irradiated by different laser wavelengths to induce MSCs to differentiate into cementoblasts, fibroblasts, and osteoblasts and therefore achieve periodontal tissue regeneration.

The therapeutic applications of optogenetics in humans still involve many hurdles that need to be addressed. In addition to the possible immunogenicity and mutagenicity of vectors, as with canonical genetic methods, the immune response that the optogenetic protein itself may cause needs to be investigated further. Many research studies have been performed to address this hurdle, such as using protein engineering to “humanize” optogenetic proteins, or the repositioning of human opsins as optogenetic proteins [29]. In addition, the very core of the matter of in vivo optogenetics is light delivery deep into the tissues. As mentioned earlier, the wavelength of light determines the penetration and distribution of light within the tissue. This process involves scattering and absorption coefficients, which are difficult to calculate accurately. Although the rapid change and uncertainty of light distribution limits the accuracy of its application, researchers have invented various algorithms for the rational selection of light sources. Furthermore, machine learning can be used to achieve optimal designs for optogenetic systems [30, 31]. Despite of its limitations, optogenetics still has significant potential for regenerative medicine. In our study, we simply used optogenetic tools to verify our hypothesis and did not create a precise design for the application of the optogenetic system in vivo. Future experiments should be carried out to create a more optimized and adaptive system. Our research demonstrated that optogenetics represented an alternative tool for precise gene expression in regenerative medicine. We not only revealed the beneficial effects of temporal regulation for *Lhx8* in bone regeneration, but also provided a novel research strategy for investigating vital factors in the process of development and regeneration. The application of optogenetic tools for fine process regulation will significantly increase the possibility of complex tissue regeneration.

## Conclusions

In the present study, we demonstrated the potential role of *Lhx8* in the process of osteogenic differentiation in BMSCs. We developed an optogenetic regulation system for *Lhx8* that promotes the osteogenic process in BMSCs by precisely regulating the expression levels of *Lhx8*, thus providing novel strategies for precision regenerative medicine.

## Supplementary Information

**Additional file 1: Table S1.** The primer sequences used in this study.

**Additional file 2: Figure S1.** Characterization of BMSCs. (a-b) The morphology of BMSCs from primary culture in passage 0 (a) and passage 2 (b). Scale bars=200μm. (c) Osteogenic differentiation was identified by staining calcified nodules with alizarin red. Scale bar=200μm. (d) Adipogenic differentiation was demonstrated by staining lipid droplets with Oil Red O reagent. Scale bar=20μm. (e) Chondrogenic differentiation was verified by alcian blue staining of the induced cartilage microsphere. Scale bar=100μm. (f) BMSCs were positive for CD29, CD44, and CD90, but negative for CD45 and CD34. All experiments were performed in triplicate.

**Additional file 3: Figure S2.** The time-specific role of *Lhx8* during the osteogenic differentiation of BMSCs *in vitro.* (a) ALP activity was detected by colorimetric assay in the supernatant of BMSCs on Day 0, 3, 7 and 14 under osteogenic induction. (b-c) The mRNA expression of osteogenic-specific genes (*OPN*, *Col-1a*) after osteogenic induction on Day 0, 3, 7, and 14. (d) The protein expression of osteogenic- specific genes (*OPN*, *Col-1a*) after osteogenic induction on Day 0, 3, 7, and 14. (e-j) Quantitative analysis of the protein expression of osteogenic-specific genes (*ALP*, *Runx2*, *OSX*, *OCN*, *OPN*, *Col-1a*) after osteogenic induction on Day 0, 3, 7, and 14. All experiments were performed in triplicate. *P<0.05, **P<0.01, ***P<0.001, ****P<0.0001 *vs.* Ctrl.

**Additional file 4: Figure S3.** Characterization of the customized optogenetic expression system for *Lhx8*. (a) Schematic representation of the LED light device *in vitro* experiment. (b) Relative mRNA expression of *Lhx8* in HeLa cells treated with different wavelengths of light (1 mW/cm^2^). The optogenetic expression was characterized by wavelength-specific responses; these showed significant activation following the application of blue light. (c) CCK-8 growth curves of BMSCs with or without exposure to blue light. Exposure to blue light did not cause significant damage to BMSCs. (d-e) Representative images and quantitative analysis of EdU staining (green) of BMSCs in the two groups. Nuclear DNA was counterstained with Hoechst. Scale bar=50μm. (f-g) The relative expression level of *Lhx8* in HeLa cells at different time points after 30min of blue light irradiation (1 mW/cm^2^). (h) The effect of different durations of blue light illumination (1 mW/cm^2^) on *Lhx8* expression in BMSCs. All experiments were performed in triplicates. **P<0.01, ***P<0.001, ****P<0.0001 *vs.* Ctrl (b), dark (c, e) or 0 min (h).

**Additional file 5: Figure S4.** The optogenetic regulation of *Lhx8* promoted bone formation in BMSCs *in vitro*. (a-c) Quantitative analysis of the protein expression of osteogenic-specific genes (*ALP*, *Runx2*, *OCN*) after osteogenic induction on days 7. All experiments were performed in triplicate. **P<0.01, ***P<0.001, ****P<0.0001 *vs.* Ctrl.

**Additional file 6: Figure S5.** Morphology observation and biocompatibility assays of PLGA scaffolds. (a) Gross appearance of the PLAG scaffold. Scale bar=1.0mm. (b-d) Representative images of PLGA scaffolds observed by SEM. Scale bars=1.0mm(b), 100μm(c) and 50μm(d). (e-f) Morphology of BMSCs seeded on PLGA scaffolds and observed by SEM. Scale bars=100μm(e) and 50μm(f). White arrows: BMSCs attached to the surface of the scaffolds. (g) CCK-8 assays were used to determine the viability of BMSCs on PLGA scaffolds and on control dishes.

**Additional file 7: **Blue light activated the expression of *Lhx8* in opto Lhx8 system *in vivo.* Relative mRNA expression of *Lhx8* in BMSCs treated with or without blue light in vivo. **P<0.01 *vs.* opto Lhx8+dark.

**Additional file 8.** Corresponding sequence in the study.

## Data Availability

All data generated or analyzed during this study are included in this published article and its supplementary information files.

## Abbreviations

*Lhx8*: LIM Homeobox 8
MSCs: Mesenchymal stem cells
*ALP*: Alkaline phosphatase
*Runx2*: Runt-related transcription factor 2
*OSX*: Osterix
*OCN*: Osteocalcin
*OPN*: Osteopontin
*Col-1*: Collagen type I
*Col-1a*: Collagen type I alpha
FKF1: Flavin-binding, kelch repeat, f-box 1
LOV: Light, oxygen or voltage
GI: GIGANTEA
FMN: Flavin mononucleotide
DPSCs: Dental pulp-derived mesenchymal stem cells
BMSCs: Bone marrow mesenchymal stem cells
PBS: Phosphate-buffered solution
CPC: Cetylpyridinium chloride
EdU: 5-Ethynyl-20-deoxyuridine
PLGA: Poly(lactic-co-glycolic acid)
DCM: Dichloromethane
NaCl: Sodium chloride
SEM: Scanning electron microscope
BV: Bone volume
TV: Total volume
BMD: Bone mineral density
EDTA: Ethylene diamine tetraacetic acid
HE: Hematoxylin and eosin
DAB: Diaminobenzidine
