# Supplementary material for: Temporal induction of Lhx8 by optogenetic control system for efficient bone regeneration
Source: Stem Cell Res Ther. 2021 Jun 10;12:339. doi: 10.1186/s13287-021-02412-8 (PMC8194135; doi:10.1186/s13287-021-02412-8)
Supplement: Supplementary file 1 — Additional file 1: Table S1. The primer sequences used in this study. [file 13287_2021_2412_MOESM1_ESM.pdf]

Table S1 Primer sequences used in the study.

**Name**	**Forward (5' to 3')**	**Reverse (5' to 3')**
Rat and human Lhx8	CTTGGCTTGCTTTGCCTGCTT	TGGAGGGTCTGTGCGTCTGG
Rat GAPDH	GCAAGTTCAACGGCACAG	GCCAGTAGACTCCACGACAT
Rat ALP	TATGGCTCACCTGCTTCACGG	GCTGTCCATTGTGGGCTCTTG
Rat Runx2	TTAGGGCGCATTCCTCATCC	GTCAGAGGTGGCAGTGTCAT
Rat OSX	CTGGGAAAAGGAGGCACAAAGA	GGGGAAAGGGTGGGTAGTCATT
Rat OPN	TGAAGAGCCAGGAGTCCGAT	GCTTTGGAACTCGCCTGACT
Rat OCN	AGCTCAACCCCAATTGTGAC	AGCTGTGCCGTCCATACTTT
Rat Col-1a	ACCTTCGCTTCCATACTCG	CACTCAGCCCTCTGTGCCT
Human GAPDH	GAGTCAACGGATTTGGTCGT	GACAAGCTTCCCGTTCTCAG
