# Supplementary figures and images for: Temporal induction of Lhx8 by optogenetic control system for efficient bone regeneration
Source: Stem Cell Res Ther. 2021 Jun 10;12:339. doi: 10.1186/s13287-021-02412-8 (PMC8194135; doi:10.1186/s13287-021-02412-8)

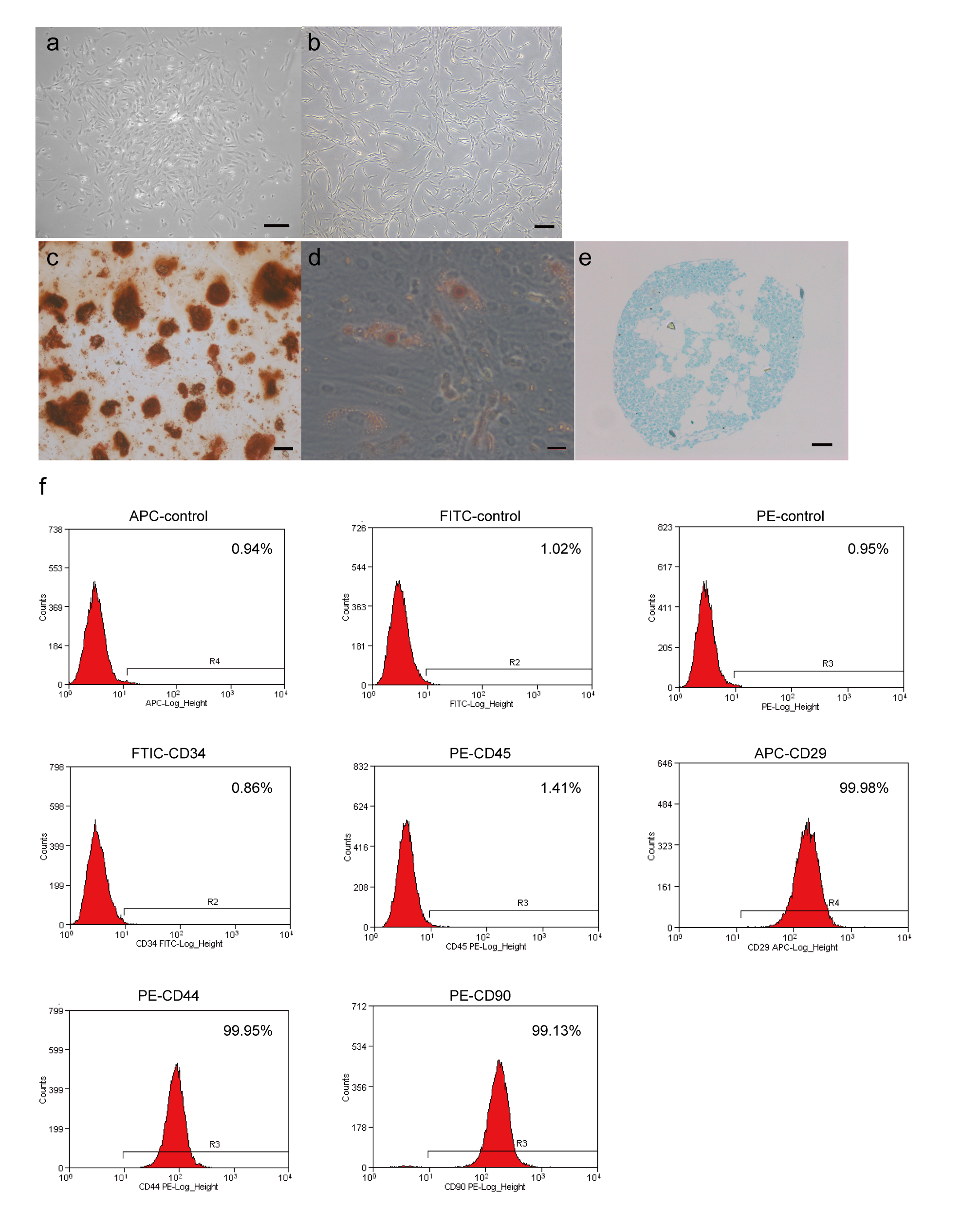

Supplement: Supplementary file 2 — Additional file 2: Figure S1. Characterization of BMSCs. (a-b) The morphology of BMSCs from primary culture in passage 0 (a) and passage 2 (b). Scale bars=200μm. (c) Osteogenic differentiation was identified by staining calcified nodules with alizarin red. Scale bar=200μm. (d) Adipogenic differentiation was demonstrated by staining lipid droplets with Oil Red O reagent. Scale bar=20μm. (e) Chondrogenic differentiation was verified by alcian blue staining of the induced cartilage microsphere. Scale bar=100μm. (f) BMSCs were positive for CD29, CD44, and CD90, but negative for CD45 and CD34. All experiments were performed in triplicate. [file 13287_2021_2412_MOESM2_ESM.tif]

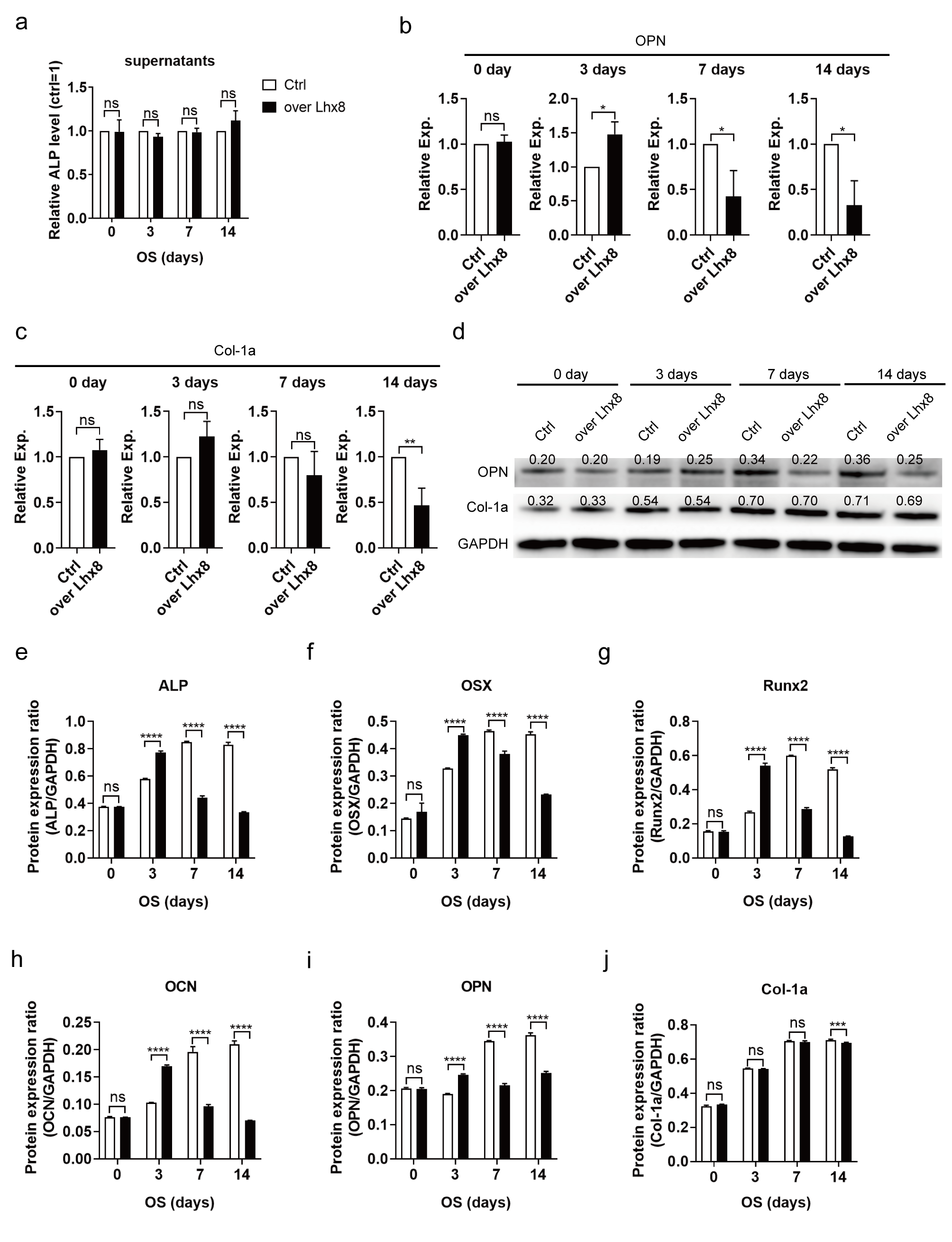

Supplement: Supplementary file 3 — Additional file 3: Figure S2. The time-specific role of Lhx8 during the osteogenic differentiation of BMSCs in vitro. (a) ALP activity was detected by colorimetric assay in the supernatant of BMSCs on Day 0, 3, 7 and 14 under osteogenic induction. (b-c) The mRNA expression of osteogenic-specific genes (OPN, Col-1a) after osteogenic induction on Day 0, 3, 7, and 14. (d) The protein expression of osteogenic- specific genes (OPN, Col-1a) after osteogenic induction on Day 0, 3, 7, and 14. (e-j) Quantitative analysis of the protein expression of osteogenic-specific genes (ALP, Runx2, OSX, OCN, OPN, Col-1a) after osteogenic induction on Day 0, 3, 7, and 14. All experiments were performed in triplicate. *P<0.05, **P<0.01, ***P<0.001, ****P<0.0001 vs. Ctrl. [file 13287_2021_2412_MOESM3_ESM.tif]

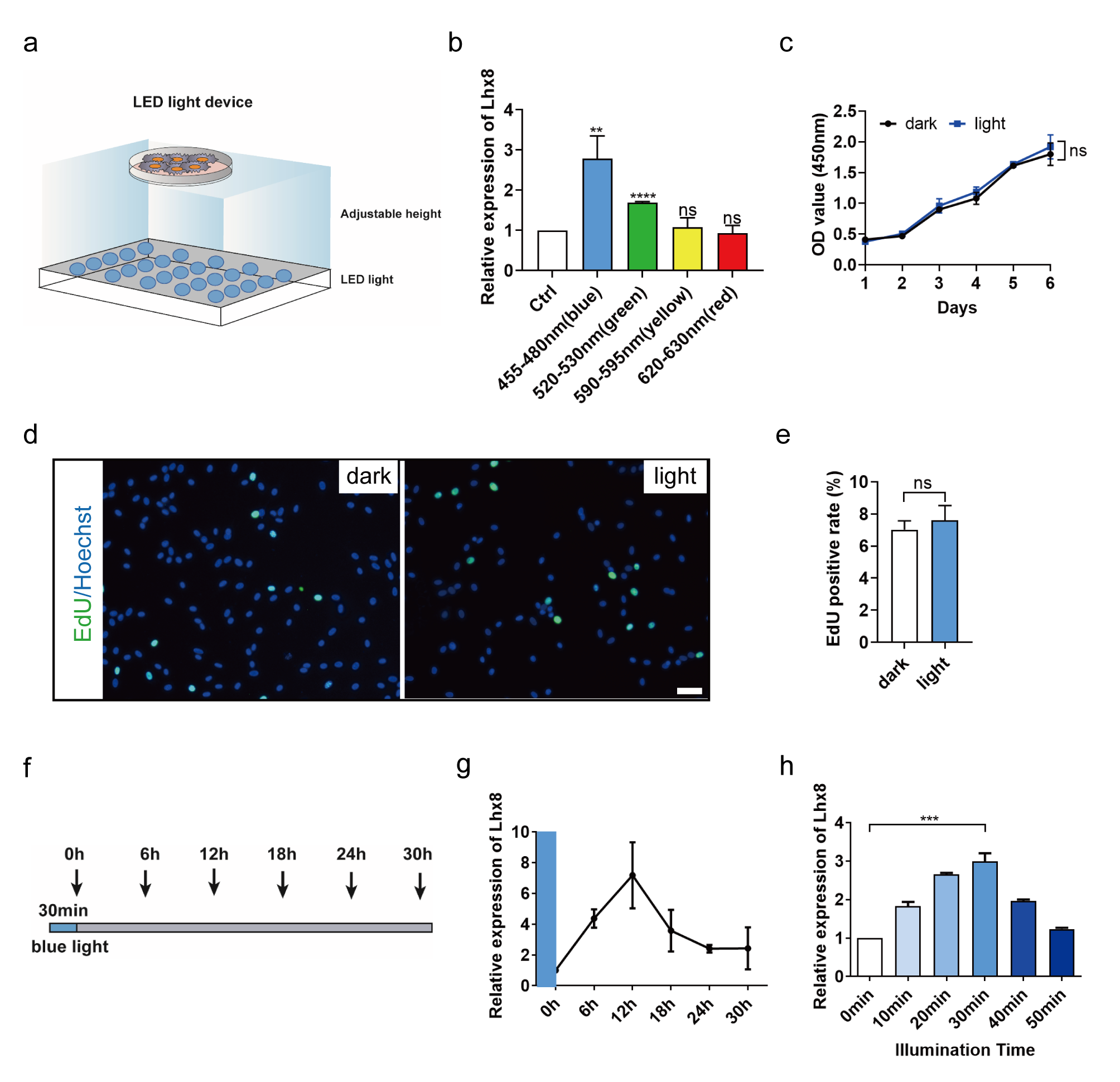

Supplement: Supplementary file 4 — Additional file 4: Figure S3. Characterization of the customized optogenetic expression system for Lhx8. (a) Schematic representation of the LED light device in vitro experiment. (b) Relative mRNA expression of Lhx8 in HeLa cells treated with different wavelengths of light (1 mW/cm2). The optogenetic expression was characterized by wavelength-specific responses; these showed significant activation following the application of blue light. (c) CCK-8 growth curves of BMSCs with or without exposure to blue light. Exposure to blue light did not cause significant damage to BMSCs. (d-e) Representative images and quantitative analysis of EdU staining (green) of BMSCs in the two groups. Nuclear DNA was counterstained with Hoechst. Scale bar=50μm. (f-g) The relative expression level of Lhx8 in HeLa cells at different time points after 30min of blue light irradiation (1 mW/cm2). (h) The effect of different durations of blue light illumination (1 mW/cm2) on Lhx8 expression in BMSCs. All experiments were performed in triplicates. **P<0.01, ***P<0.001, ****P<0.0001 vs. Ctrl (b), dark (c, e) or 0 min (h). [file 13287_2021_2412_MOESM4_ESM.tif]

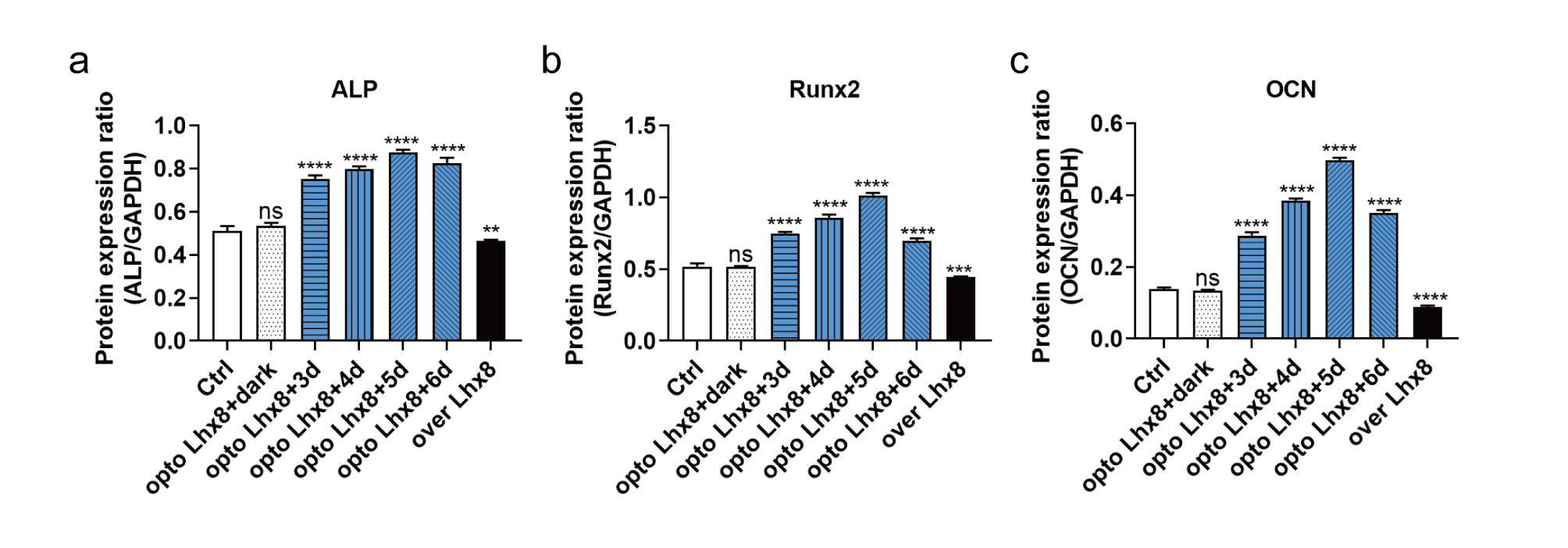

Supplement: Supplementary file 5 — Additional file 5: Figure S4. The optogenetic regulation of Lhx8 promoted bone formation in BMSCs in vitro. (a-c) Quantitative analysis of the protein expression of osteogenic-specific genes (ALP, Runx2, OCN) after osteogenic induction on days 7. All experiments were performed in triplicate. **P<0.01, ***P<0.001, ****P<0.0001 vs. Ctrl. [file 13287_2021_2412_MOESM5_ESM.tif]

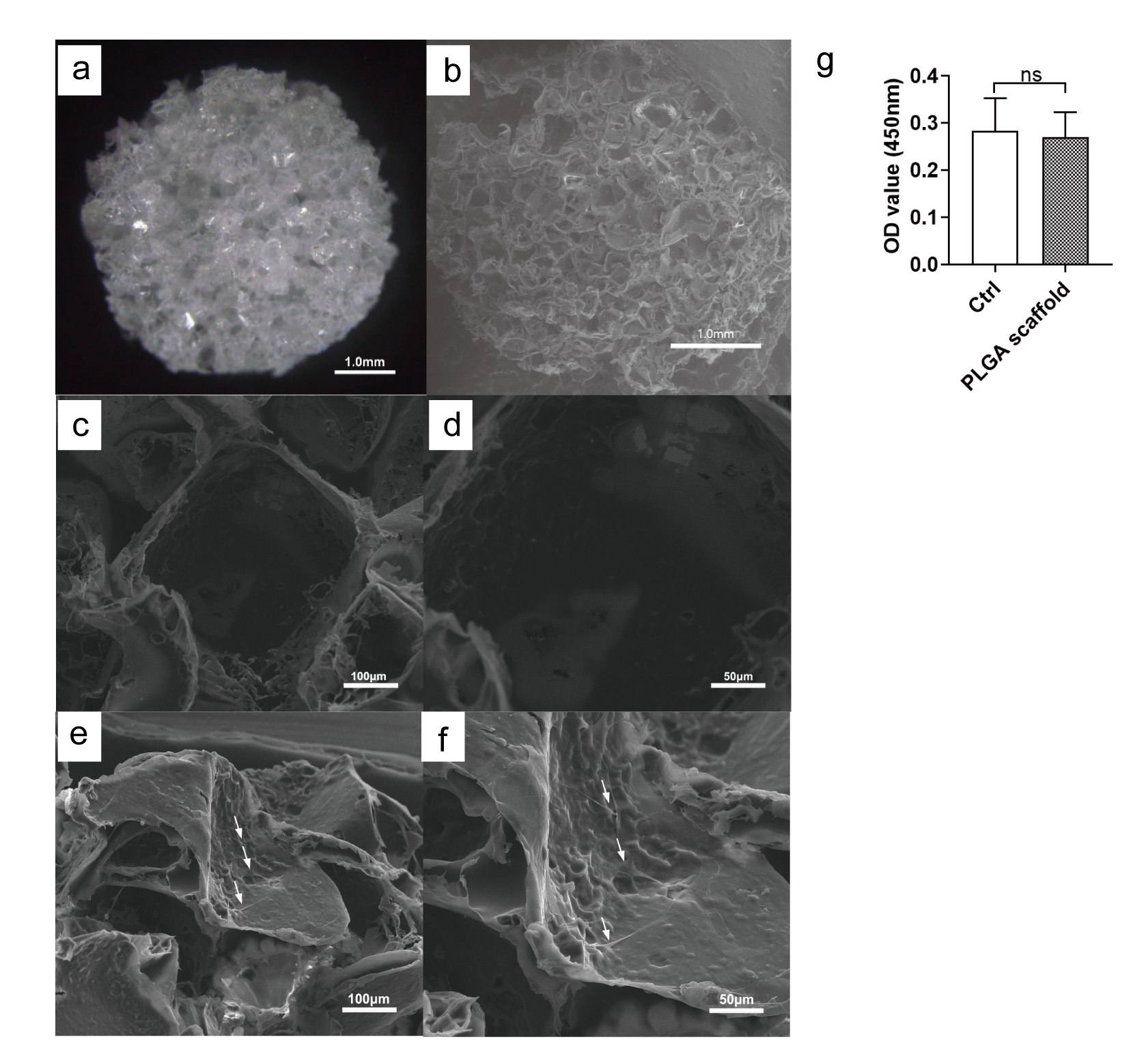

Supplement: Supplementary file 6 — Additional file 6: Figure S5. Morphology observation and biocompatibility assays of PLGA scaffolds. (a) Gross appearance of the PLAG scaffold. Scale bar=1.0mm. (b-d) Representative images of PLGA scaffolds observed by SEM. Scale bars=1.0mm(b), 100μm(c) and 50μm(d). (e-f) Morphology of BMSCs seeded on PLGA scaffolds and observed by SEM. Scale bars=100μm(e) and 50μm(f). White arrows: BMSCs attached to the surface of the scaffolds. (g) CCK-8 assays were used to determine the viability of BMSCs on PLGA scaffolds and on control dishes. [file 13287_2021_2412_MOESM6_ESM.tif]

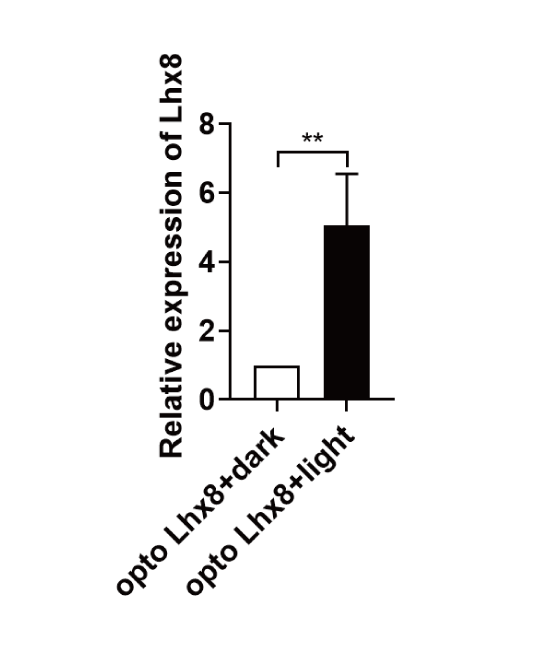

Supplement: Supplementary file 7 — Additional file 7: Blue light activated the expression of Lhx8 in opto Lhx8 system in vivo. Relative mRNA expression of Lhx8 in BMSCs treated with or without blue light in vivo. **P<0.01 vs. opto Lhx8+dark. [file 13287_2021_2412_MOESM7_ESM.tif]
