# Supplementary material for: Temporal induction of Lhx8 by optogenetic control system for efficient bone regeneration
Source: Stem Cell Res Ther. 2021 Jun 10;12:339. doi: 10.1186/s13287-021-02412-8 (PMC8194135; doi:10.1186/s13287-021-02412-8)
Supplement: Supplementary file 8 — Additional file 8. Corresponding sequence in the study. [file 13287_2021_2412_MOESM8_ESM.pdf]

Corresponding sequence in the study

5×GAL4 UAS

AGTTAGGCAGGGATATTCACCATTATCGTTTCAGACCCACCTCCCAACCCCGAGGGG
ACCCGACAGGCCCGAAGGAATAGAAGAAGAAGGTGGAGAGAGAGACAGAGACAGA
TCCATTCGATTAGTGAACGGATCTCGACGGTATCGATCACGAGACTAGCCTCGACCAT
CGATACGGAGTACTGTCCTCCGAGCGGAGTACTGTCCTCCGACTCGAGCGGAGTACT
GTCCTCCGATCGGAGTACTGTCCTCCGCGAATTCCGGAGTACTGTCCTCCGAAGACGC
TAGCGGGGGGCTATAAAAGGGGGTGGGGGCGTTCGTCCTCACTCTAGATCTGCGATC
TAAGTAAGCTTGGCATTCCGGTACTGTTGGTAAAGCCACCTTAATTAATCTCGACGGT
ATCGGTTAACTTTTAAAAGAAAAGGGGGGATTGGGGGGTACAGTGCAGGGGAAAGA
ATAGTAGACATAATAGCAACAGACATACAAACTAAAGAATTACAAAAACAAATTAC
AAAAATTCAAAATTTTATCCATCACGAGACTAGCCTCGAGATTTAAATTTAATTAAGG
GGCCATGTATTGGAAGAGCGATCAGATGTTTGTGTGTAAGCTGGAGGGAAAGGAAGT
GCCGGAGCTGGCGGTTCCCCGCGAGAAGTGCCCCGGGCTCATGTCGGAGGAGTGCGG
GCGGCCTGCAGCCCTGGCGGCCGGGAGGACCCGCAAAGGCGCTGGGGAAGAAGGAC
TGGTGAATC

GI-GAL4DBD

TTAATTAACCATGGCTAGTTCATCTTCATCTGAGAGATGGATCGATGGTCTTCAGTTCT
CTTCCTTGTTATGGCCTCCGCCACGAGATCCTCAACAACATAAGGATCAAGTCGTTGC
TTATGTTGAATATTTTGGTCAATTTACATCAGAGCAATTCCCAGATGACATTGCTGAG
TTGGTCCGGCATCAGTATCCATCAACCGAGAAGCGACTTTTGGACGATGTGCTGGCG
ATGTTTGTCCTTCATCATCCGGAGCATGGTCATGCAGTCATTCTTCCAATCATTTCATG
TCTTATTGATGGCTCGTTGGTGTACAGCAAGGAAGCTCATCCGTTTGCCTCTTTCATAT
CTTTAGTTTGCCCAAGTAGTGAGAATGACTATTCGGAGCAATGGGCTTTGGCATGTGG
AGAAATCCTTCGCATTTTGACTCATTACAACCGTCCCATTTATAAAACTGAGCAGCAA
AATGGAGATACAGAGAGAAATTGTCTGAGCAAAGCTACAACTAGTGGTTCTCCGACT
TCAGAGCCTAAGGCTGGATCACCAACACAGCATGAAAGGAAACCTTTAAGGCCTTTG
TCTCCATGGATCAGTGATATACTACTTGCTGCTCCTCTTGGTATAAGAAGTGACTATTT
CCGATGGTGTAGTGGTGTAATGGGTAAATATGCTGCTGGAGAGCTCAAGCCGCCAAC
CATTGCTTCTCGAGGATCTGGTAAACATCCTCAACTGATGCCTTCAACCCCAAGATGG

GCTGTTGCTAATGGAGCTGGTGTCATACTGAGTGTTTGTGATGATGAAGTTGCTCGAT

ATGAGACTGCTACGCTGACAGCGGTCGCTGTCCCTGCACTTCTTCTTCCTCCGCCAAC

GACATCCTTAGATGAGCATCTAGTTGCTGGCCTTCCAGCTCTTGAACCATATGCACGT

TTGTTTCATAGATACTATGCCATTGCAACTCCAAGTGCTACGCAGAGACTTCTTCTTG

GACTCTTAGAAGCACCACCGTCGTGGGCTCCAGATGCACTTGATGCTGCTGTACAGCT

TGTGGAACTCCTTCGAGCTGCTGAAGATTATGCATCTGGTGTAAGGCTACCCAGGAAC

TGGATGCATTTGCACTTCTTGCGGGCTATAGGAATTGCTATGTCTATGAGGGCAGGTG

TTGCTGCTGATGCTGCAGCCGCTTTGCTTTTCCGCATACTCTCACAGCCGGCACTGCTT

TTTCCTCCGCTAAGTCAAGTTGAGGGAGTAGAAATTCAGCACGCGCCTATTGGTGGCT

ACAGTTCAAATTACAGAAAACAGATAGAAGTTCCTGCAGCAGAAGCAACCATTGAAG

CCACTGCCCAAGGAATTGCCTCAATGCTTTGTGCTCATGGTCCTGAAGTTGAGTGGAG

AATTTGCACTATATGGGAAGCTGCTTATGGTTTGATCCCTTTAAATTCTTCGGCGGTTG

ATCTTCCCGAAATCATAGTTGCTACCCCACTGCAACCTCCTATCTTGTCATGGAATTTA

TACATTCCACTCCTCAAAGTACTTGAATATCTTCCACGGGGGAGTCCTTCGGAAGCAT

GCTTGATGAAAATATTTGTTGCCACTGTGGAAACAATACTCAGTAGAACTTTTCCGCC

TGAATCTTCCAGGGAACTAACCAGAAAAGCTAGATCGAGTTTTACCACAAGATCAGC

GACCAAAAATCTTGCTATGTCTGAGCTTCGTGCTATGGTCCATGCTCTCTTTTTAGAAT

CATGCGCTGGTGTGGAATTAGCTTCACGCCTACTTTTTGTTGTGTTGACTGTATGTGTT

AGCCATGAAGCACAGTCTAGTGGTAGCAAGAGACCGAGAAGTGAATATGCTAGTACT

ACTGAAAATATTGAGGCGAATCAACCTGTATCTAACAATCAAACTGCTAACCGTAAA

AGTAGGAATGTCAAGGGACAGGGACCTGTGGCAGCATTTGATTCATACGTTCTTGCT

GCTGTTTGTGCTCTTGCCTGTGAGGTTCAGCTGTATCCTATGATCTCTGGTGGGGGA

ACTTTTCCAATTCTGCCGTGGCTGGAACTATTACAAAGCCTGTAAAGATAAATGGGTC

ATCTAAAGAGTATGGAGCTGGGATTGACTCGGCAATTAGTCATACGCGCCGAATTTT

GGCAATCCTAGAGGCACTCTTTTCATTAAAACCATCTTCTGTGGGGACTCCATGGAGT

TACAGTTCTAGTGAGATAGTTGCTGCGGCCATGGTTGCAGCTCATATTTCCGAACTGT

TCAGACGTTCAAAGGCCTTGACGCATGCATTGTCTGGGTTGATGAGATGTAAGTGGG

ATAAGGAAATTCATAAAAGAGCATCATCATTATATAACCTCATAGATGTTCACAGCA

AAGTTGTTGCCTCCATTGTTGACAAAGCTGAACCCTTGGAAGCCTACCTTAAGAATAC

ACCGGTTCAGAAGGATTCTGTGACCTGTTTAAACTGGAAACAAGAGAACACATGTGC

AAGCACCACATGCTTTGATACAGCGGTGACATCCGCCTCAAGGACTGAAATGAATCC

AAGAGGAAACCATAAGTATGCTAGACATTCAGATGAAGGCTCAGGAAGACCCTCAG

AGAAGGGTATCAAAGATTTCCTCTTGGATGCTTCTGATCTAGCGAATTTCCTCACAGC

TGATAGACTCGCAGGGTTCTATTGTGGTACACAAAAGCTTTTGAGGTCAGTGCTTGCA

GAGAAACCGGAGCTGTCTTTCTCCGTTGTTTCACTGTTATGGCACAAACTGATTGCTG

CTCCTGAAATCCAGCCCACCGCAGAAAGCACCTCTGCGCAACAAGGATGGAGACAGG

TTGTTGATGCGCTATGCAATGTCGTATCTGCAACGCCAGCGAAAGCAGCAGCAGCAG

TTGTCCTTCAGGCTGAAAGGGAGTTGCAGCCTTGGATCGCCAAAGATGATGAAGAAG

GCCAAAAAATGTGGAAAATCAACCAACGGATAGTCAAAGTGTTGGTGGAACTCATGC

GCAATCATGACAGGCCTGAGTCACTGGTGATTCTCGCAAGTGCATCAGATCTTCTTCT

GCGGGCAACTGATGGAATGCTTGTTGATGGAGAAGCTTGTACATTACCTCAACTTGA

GCTACTTGAAGCCACGGCAAGAGCAATACAGCCGGTGCTAGCTTGGGGGCCATCTGG

ACTAGCAGTGGTCGACGGTTTATCCAATCTATTGAAGTGTCGTCTACCAGCAACAATA

CGGTGCCTTTCACACCCAAGTGCACACGTACGTGCCTTAAGCACGTCAGTACTACGTG

ATATCATGAACCAAAGCTCCATACCCATCAAAGTAACTCCAAAACTGCCAACAACAG

AGAAGAACGGAATGAATAGTCCGTCCTATCGATTCTTCAACGCCGCCTCAATAGACT

GGAAAGCCGATATCCAAAACTGTTTAAACTGGGAAGCTCACAGCTTGCTCTCCACAA

CTATGCCTACTCAGTTTCTCGACACTGCGGCTCGGGAACTCGGCTGTACTATATCCTT

GTCCCAAGCGGCCGCAAGTGCTGGTAAGCTACTGTCTTCTATCGAACAAGCATGCGA

TATTTGCCGACTTAAAAAGCTCAAGTGCTCCAAAGAAAAACCGAAGTGCGCCAAGTG

TCTGAAGAACAACTGGGAGTGTCGCTACTCTCCCAAAACCAAAAGGTCTCCGCTGAC

TAGGGCACATCTGACAGAAGTGGAATCAAGGCTAGAAAGACTGGAACAGCTATTTCT

ACTGATTTTTCCTCGAGAAGACCTTGACATGATTTTGAAAATGGATTCTTTACAGGAT

ATAAAAGCATTGTTAACAGGATTATTTGTACAAGATAATGTGAATAAAGATGCCGTC

ACAGATAGATTGGCTTCAGTGGAGACTGATATGCCTCTAACATTGAGACAGCATAGA

ATAAGTGCGACATCATCATCGGAAGAGAGTAGTAACAAAGGTCAAAGACAGTTGACT

GTATCGCCGTAATTAATTAA

LOV-VP16

ACCATGGCTTACCCATACGATGTTCCAGATTACGCTGCGAGAGAACATGCGATCGGA

GAAGCCACCGGAAAACGAAAGAAACGCGGCAGAGTTGAAGAAGCAGAAGAATACTG

TAACGATGGAATCGAAGAACAAGTAGAGGATGAGAAGCTTCCGTTAGAGGTTGGGAT

GTTCTATTACCCAATGACTCCGCCTTCGTTCATTGTTTCCGATGCTCTGGAGCCAGATT

TTCCTTTGATCTATGTCAACAGAGTCTTCGAAGTCTTCACTGGCTATCGTGCCGATGA

AGTTCTTGGTCGTAACTGTCGATTCCTACAGTACAGAGATCCTCGAGCTCAAAGGCGT

CACCCATTGGTTGATCCTGTGGTTGTATCTGAGATTAGGAGATGTCTTGAAGAAGGTA

TTGAATTCCAAGACGAGCTTCTTAATTTCAGAAAAGATGGTACTCCTTTGGTTAACAG

ACTACGGCTTGCTCCAATACGTGACGATGATGGAACCATTACACACGTAATTGGGAT

ACAGGTCTTCTCTGAAACGGGTACCGAAAGCGGCGGGCCGGCCGACGCCCTTGACGA

TTTTGACTTAGACATGCTCCCAGCCGATGCCCTTGACGACTTTGACCTTGATATGCTG

CCTGCTGACGCTCTTGACGATTTTGACCTTGACATGCTCCCCGGGTAA
